# Searching for the Virulence-contributing Genes of the *Magnaporthe oryzae* by Transcriptome Analysis

**DOI:** 10.3390/pathogens13020105

**Published:** 2024-01-25

**Authors:** Jitao Hu, Linying Li, Yuqing He, Gaojie Hong, Chi Zhang

**Affiliations:** State Key Laboratory for Managing Biotic and Chemical Treats to the Quality and Safety of Agro-Products, Key Laboratory of Biotechnology in Plant Protection of MOA of China and Zhejiang Province, Institute of Virology and Biotechnology, Zhejiang Academy of Agricultural Sciences, Hangzhou 310021, China; hjt2784156330@163.com (J.H.);

**Keywords:** *Magnaporthe oryzae*, transcriptome, salicylic acid, abscisic acid, sakuranetin

## Abstract

*Magnaporthe oryzae* is a fungal pathogen that causes rice blast. Plant metabolites such as plant hormones and phytoalexin can promote or inhibit the rice blast infection. To study the effect of plant metabolites on *M. oryzae*, we selected salicylic acid (SA), abscisic acid (ABA), and a phytoalexin sakuranetin to treat *M. oryzae* grown on the medium. Through the analysis of transcriptome data, 185 and 38 genes, 803 and 156 genes, and 1525 and 428 genes were up- or down-regulated after SA, ABA, or sakuranetin treatment. Among these differentially expressed genes (DEGs), most of them were annotated to the cellular process and metabolic process in the biological process category and binding and catalytic activity in the molecular function category by GO analysis. According to KEGG pathway analysis, metabolism is the pathway with the highest number of DEGs, and the main enriched pathway is carbohydrate, lipid, and amino acid metabolism. In addition, we also found two ABA-induced up-regulated genes that may contribute to *M. oryzae* infection from the transcriptome data. We verified their expressions in *M. oryzae* that infected rice.

## 1. Introduction

*Magnaporthe oryzae* is a fungal pathogen that causes rice blast, one of the most severe diseases of rice. The infection of rice blast can result in a loss of 10–30% of rice production, and in a few severe cases, it can even lead to the total loss of rice production [1]. *M. oryzae* is a hemibiotrophic pathogen. In the early stage of infection, the spores germinate and invade plant cells, and the hyphae expand within the cells and absorb nutrients from living cells. As the fungus rapidly grows, the plant tissue begins to die, and the fungus switches to necrotrophic growth [2]. As a plant pathogen, its growth state is affected by various plant secondary metabolites.

Plant hormones regulate every aspect of plant life from germination to death. The interaction between plants and pathogens is also regulated by these hormones [3]. Salicylic acid (SA) is one of the most important hormones that contribute to plant immunity. Numerous studies have shown that SA can regulate systemic acquired resistance in plants [4,5]. It can be long-distance transported in plants and make uninfected plant tissues resistant to disease [6]. When the SA hydroxylases are knocked out, the SA will accumulate in rice, and the basal resistance to rice blast will increase [7]. In addition to plant hormones like salicylic acid that promote disease resistance, some plant hormones negatively regulate plant immunity. Abscisic acid (ABA) is a pleiotropic plant hormone that refers to leaf senescence, bud dormancy, seed germination, osmotic regulation, and growth inhibition [8]. When *M. oryzae* invades rice, ABA can increase plant susceptibility and accelerate pathogenesis. In addition, exogenous ABA promotes spore germination and appressoria formation [9]. Therefore, exploring the regulation of these plant hormones on the growth of *M. oryzae* is helpful to understand the infection mechanism further.

Besides the plant hormones, plants can also synthesize some secondary metabolites that directly fight against pathogens called phytoalexins. Phytoalexins are the molecules produced by plants in response to infection or stress. Unlike the plant hormones that mainly regulate the physiological processes of plants, phytoalexins usually act directly on the pathogens and exhibit antibacterial, antifungal, or antiviral activity [10]. The phytoalexins in rice belong to the classes of diterpenes, flavonoids, and phenylamides [11]. Though there are various phytoalexins in rice, sakuranetin is the only phytoalexin that belongs to flavonoids, which is catalyzed from the methylation of the hydroxyl group at position 7 of naringenin [12]. Sakuranetin accumulated in *M. oryzae*-infected regions, and treatment of sakuranetin could significantly inhibit fungal growth [13]. Sakuranetin is an effective phytoalexin. However, its molecular mechanism of inhibiting rice blast is still unclear.

Exploring the transcriptional changes of *M. oryzae* genes after treatment with different plant metabolites helps understand the pathogenic mechanism of rice blast, which can provide a reference for the prevention and control of rice blast and ultimately ensure food security. In this study, we treated the *M. oryzae* with plant hormones SA, ABA, or phytoalexin sakuranetin. We compared the effects of these metabolites on the growth and pathogenicity of *M. oryzae* and analyzed the transcriptome of different treatments.

## 2. Materials and Methods

### 2.1. Fungal Strain and Plant Growth Conditions

The *Magnaporthe oryzae* wild-type strain Guy11 was used for inoculation and treatments. *M. oryzae* was cultured on CM (10 g/L glucose, 2 g/L peptone, 1 g/L yeast extract, 1 g/L casamino acids, 0.1% (*v*/*v*) trace elements, 0.1% (*v*/*v*) vitamin supplement, 6 g/L NaNO_3_, 0.5 g/L KCI, 0.5 g/L MgSO_4_, 1.5 g/L KH_2_PO_4_, pH 6.5) [14] at 25 °C under a light–dark cycle (16 h–8 h). For SA, ABA, or sakuranetin treatment, the metabolites were added into the CM medium with a concentration of 100 μM. After 10 days, the diameter of the fungus was counted.

Rice (*Oryza sativa* cv. Nipponbare) was grown at 28 °C under a light–dark cycle (16 h–8 h). For rice blast inoculation, 3 to 4 weeks of rice seedlings were used.

### 2.2. Infection of Rice Blast Fungus

Blast fungus was grown for two weeks to produce spores. Spores were collected by water and adjusted to 1 × 10^6^ per milliliter. The resistance of rice plants to *M. oryzae* was evaluated by the punch inoculation. Rice leaves were lightly wounded with a mouse ear punch, and 10 μL of spore suspension was added to the wound. To evaluate the influence of metabolites on pathogenicity, 100 μM SA, ABA, or sakuranetin were added into the spore suspension, and 10 μL spore suspension was dripped onto the wounded leaves. The lesion area of rice leaves was measured after 5 days post-inoculation. The inoculated plants were returned to the growth chamber. Areas of lesions were assessed 6 or 7 days after inoculation.

### 2.3. Measurement of Relative Fungal Growth

A total 2 cm of infected leaves centered on the lesion were cut, and total DNA was extracted using a cetyltrimethylammonium bromide method. The relative expression of *M. oryzae* Pot2 gene and the rice ubiquitin gene were measured by real-time PCR. The primer sequences of Pot2 and ubiquitin and the computing formula of relative fungal growth are as described [15].

### 2.4. RNA Isolation and Library Preparation

The TRIzol reagent (Invitrogen, Carlsbad, CA, USA) was used for total RNA extraction according to the manufacturer’s protocol. RNA was purified and quantificated using the NanoDrop 2000 spectrophotometer (Thermo Scientific, Waltham, MA, USA). RNA integrity was assessed using the Agilent 2100 Bioanalyzer (Agilent Technologies, Santa Clara, CA, USA). Then, the libraries were constructed using VAHTS Universal V6 RNA-seq Library Prep Kit (Vazyme, Nanjing, China) according to the manufacturer’s instructions. The transcriptome sequencing and analysis were conducted by OE Biotech Co., Ltd. (Shanghai, China).

### 2.5. RNA Sequencing

The libraries were sequenced on an Illumina Novaseq 6000 platform, and 150 bp paired-end reads were generated. Raw reads of fastq format were firstly processed using fastp, and the low-quality reads were removed to obtain the clean reads. The clean reads were mapped to the reference genome using HISAT2. FPKM of each gene was calculated, and HTSeq-count obtained the read counts of each gene. PCA analysis was performed using R (v 3.2.0) to evaluate the biological duplication of samples.

### 2.6. DEG Analysis

Differentially expressed gene (DEG) analysis was performed using the DESeq2. Q value < 0.05 and fold change > 2 or fold change < 0.5 was set as the threshold for significant DEGs. Hierarchical cluster analysis of DEGs was performed using R (v 4.0.3) to demonstrate the expression pattern of genes in different groups and samples. The radar map of the top 30 genes was drawn to show the expression of up-regulated or down-regulated DEGs using an R packet grader. GO enrichment and KEGG pathway enrichment analyses of DEGs were, respectively, performed using R (version 4.0.3) based on the hypergeometric distribution. R (v 4.0.3) was used to draw the column diagram, the chord diagram, and the bubble diagram of the significant enrichment term. Bioinformatic analysis was performed using the OECloud tools at https://cloud.oebiotech.com/task/ accessed on 1 December 2023. The reference *M. oryzae* strain is 70-15/ATCC MYA-4617/FGSC 8958 (taxon ID 242507). The GO database was downloaded from https://geneontology.org/docs/download-ontology/ (accessed on 19 December 2023). The up-to-date annotation set for *M. oryzae* was downloaded from https://www.ebi.ac.uk/QuickGO/annotations?taxonId=242507&taxonUsage=exact (accessed on 19 December 2023). The KEGG pathway information of *M. oryzae* was referenced from https://www.kegg.jp/kegg-bin/search_pathway_text?map=mgr (accessed on 19 December 2023). The gene expression data in this study is in Table S1. The annotation information of genes from QuickGo is in Table S2. The genes’ expression changes of ABA treatment vs. mock treatment, SA treatment vs. mock treatment and sakuranetin treatment vs. mock treatment are in Tables S3–S5.

### 2.7. Quantitative Real-Time PCR

Total RNA extraction and complementary DNA synthesis were performed according to the manufacturer’s protocol of TRIGene reagent (GenStar) and HiScript II Q RT SuperMix (Vazyme, China), respectively. The cDNA was used for quantitative analysis using SYBR Green PCR Mastermix (Genstar, Beijing, China). The primer pairs of the DEGs were designed using Primer Premier 5.0 software (Table S6). Quantitative real-time PCR was performed using a QuantStudio 6 Flex Real-Time PCR System (Applied Biosystems, Waltham, MA, USA). The RT-qPCR conditions were as follows: 95 °C for 3 min; 40 cycles of 95 °C for 15 s, 60 °C for 15 s, and 72 °C for 20 s. The expression levels of the DEGs were calculated using the comparative Ct method (2^−∆∆Ct^ method). The housekeeping gene *MoActin* was used as internal reference gene. Each experiment was performed with three biological replications.

## 3. Results

### 3.1. Sakuranetin Inhibits the Growth of M. oryzae

**Figure 1:**
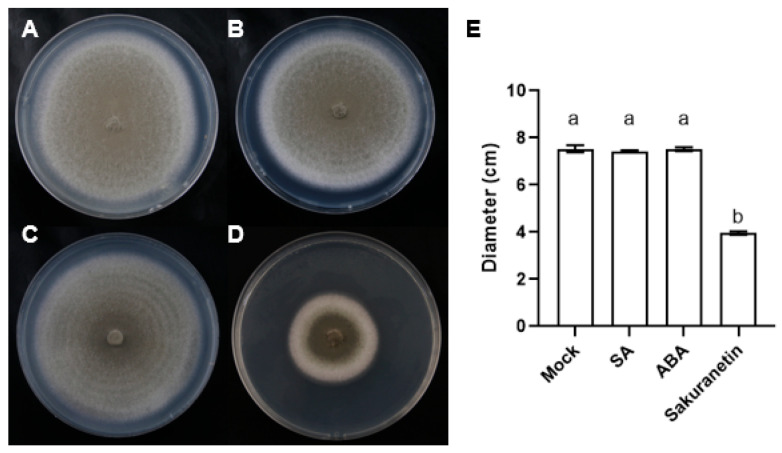
Growth of *M. oryzae* in different treatments. (**A**–**D**) Top view of *M. oryzae* on the medium containing DMSO (**A**), SA (**B**), ABA (**C**), or sakuranetin (**D**) in 10 days post inoculation. (**E**) The hyphae diameter statistics in (**A**–**D**). Letters indicate significant difference assessed by one-way ANOVA, *p* < 0.05. This is a representative experiment that was repeated three times with similar results.

The plant hormones SA, ABA, and phytoalexin sakuranetin were added into the CM medium with a concentration of 100 μM. After 10 days, the diameter of the fungus was counted, and it was found that the diameter of fungus expansion on the medium containing sakuranetin was much smaller than that in the control group and the SA or ABA treatment groups (Figure 1). This result shows that the growth of *M. oryzae* was inhibited by the sakuranetin treatment, while the plant hormones did not affect the growth of *M. oryzae* directly.

### 3.2. Effects of Sakuranetin, SA, and ABA on the Pathogenicity of M. oryzae

**Figure 2:**
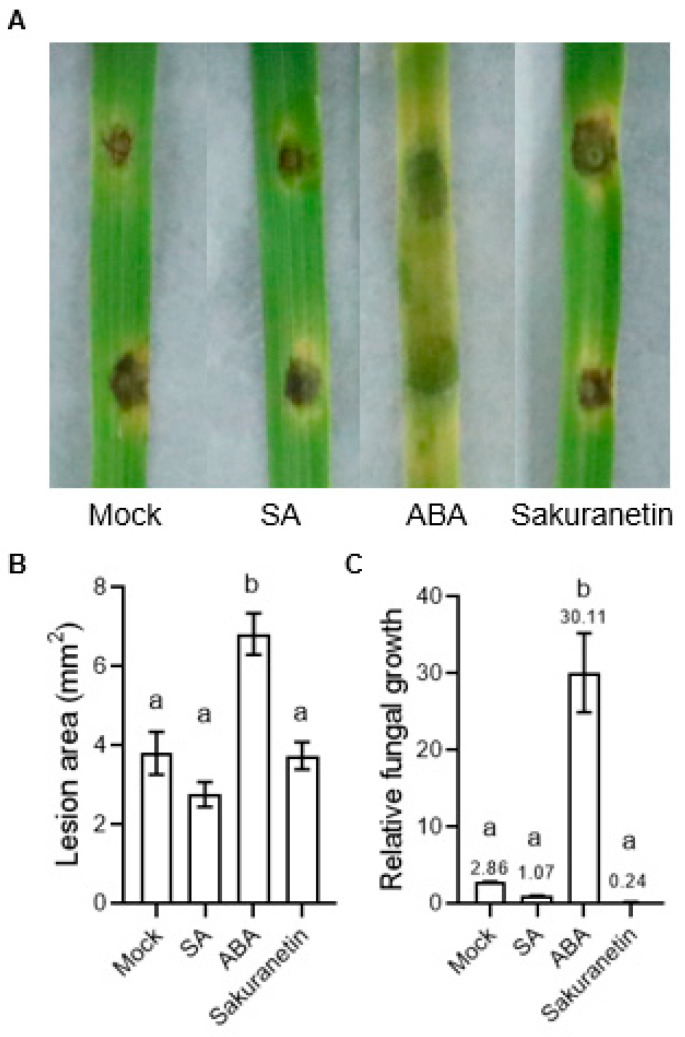
Lesion area and relative fungal growth biomass of *M. oryzae* under different treatment. (**A**) Photograph of *M. oryzae*-infected rice. (**B**) Statistics of lesion area in different treatment groups. (**C**) Relative fungal growth biomass of different treatment groups. The number on the column is the mean value detected by PCR. Letters indicate significant difference assessed by one-way ANOVA, *p* < 0.05. This is a representative experiment that was repeated three times with similar results.

To evaluate the influence of SA, ABA, and sakuranetin on the pathogenicity of *M. oryzae*, 3 to 4 weeks of rice seedlings were inoculated with the spores of *M. oryzae* by punch inoculation. The result showed that there is no significant difference in the lesion area between the mock, SA, and sakuranetin treatments. However, the ABA treatment showed a larger lesion area, and the leaves turned yellow significantly (Figure 2A,B). Besides the lesion area, the relative fungal growth biomass was also detected, and it was found that the amount of fungus on the ABA-treated leaves was significantly higher than in the other three groups (Figure 2C).

### 3.3. DEG Analysis of the M. oryzae with SA, ABA, or Sakuranetin Treatment

**Figure 3:**
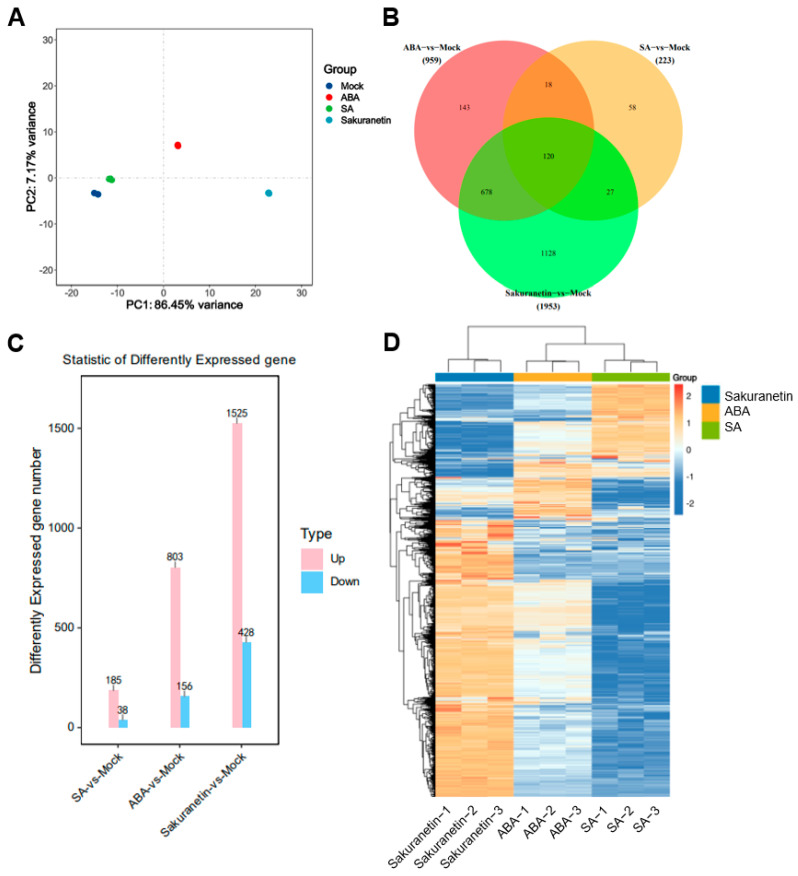
Differentially expressed genes of *M. oryzae* treated with three plant metabolites. (**A**) Principal component analysis (PCA) of DEGs under mock, SA, ABA, and sakuranetin treatments. (**B**) Common and specific DEGs between different groups. (**C**) The number of genes that were significantly up-regulated and down-regulated in different groups. (**D**) Clustering heatmap of the DEGs in different groups.

Since plants are a complex system, direct detection of the transcriptome of pathogens on plants can be interfered with by various other substances. To investigate the effect of these plant metabolites on the transcription of *M. oryzae* genes after 10-day growth on the medium with SA, ABA, or sakuranetin, the RNA of *M. oryzae* was extracted, and the transcriptome was analyzed. To assess the differences between different treatment groups and the consistency of different biological replicates within the same treatment group, principal component analysis (PCA) was performed on the transcriptome data. The PCA data show that there were significant differences among the four groups, with the contribution values of PC1 and PC2 being 86.5% and 7.17%, respectively. In the PC2 axis, the mock, SA, and sakuranetin treatment groups were mainly distributed negatively, while the ABA treatment group was distributed positively (Figure 3A). This result showed that there were obvious differences among the four groups, among which the ABA and sakuranetin groups were significantly different from the mock group, while the SA group was closer to the mock group. Then, the total number of up-regulated and down-regulated genes in the three treatment groups compared with the control group was counted. Among these differentially expressed genes, 58, 143, and 1128 genes were specifically expressed in response to SA, ABA, and sakuranetin treatments, respectively, while 120 genes with differential expression were common to all three treatments (Figure 3B). Compared with the control group, 185 genes were up-regulated and 38 genes were down-regulated after SA treatment, 803 genes were up-regulated and 156 genes were down-regulated after ABA treatment, and 1525 genes were up-regulated and 428 genes were down-regulated after sakuranetin treatment (Figure 3C,D). The sequencing data were submitted to GEO under the following accession number: GSE248232.

### 3.4. GO Enrichment Analysis of the M. oryzae with Plant Metabolites Treatment

**Figure 4:**
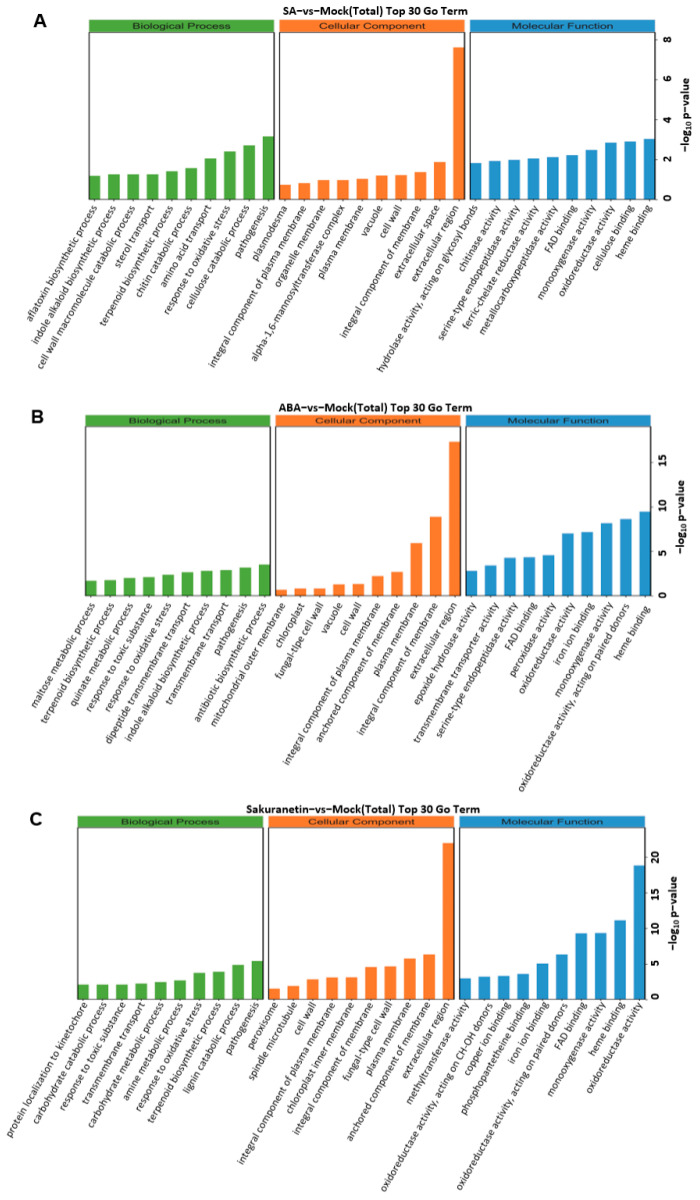
The top 30 terms of GO enrichment analysis. Terms in the three categories with more than 2 corresponding differential genes were screened, and 10 terms were ranked from largest to smallest according to the −log10 *p* value corresponding to each term. The classification of total DEGs, including up- and down-regulated genes of SA treatment (**A**), ABA treatment (**B**), and sakuranetin treatment (**C**).

The DEGs of the three treatments were divided into three categories: biological process, cellular components, and molecular function. We screened GO terms with more than two corresponding differential genes in three categories and selected the top 10 entries in each category. In the biological process category, DEGs annotated to the antibiotic biosynthetic process were enriched in the ABA treatment group. In the cellular component category, DEGs annotated to extracellular were significantly enriched in three groups (Figure 4).

### 3.5. KEGG Enrichment Analysis of the M. oryzae with Plant Metabolite Treatment

**Figure 5:**
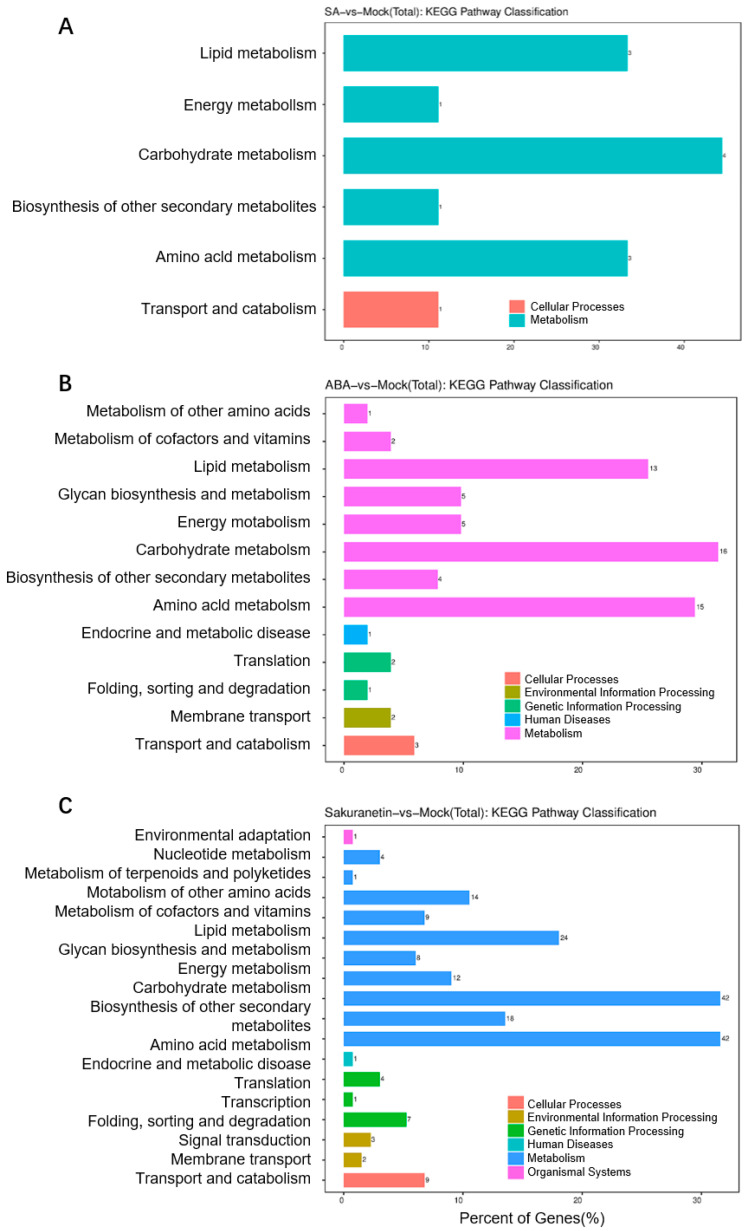
The KEGG pathway classification of three groups. The classification of total DEGs, including up- and down-regulated genes of SA treatment (**A**), ABA treatment (**B**), and sakuranetin treatment (**C**).

**Figure 6:**
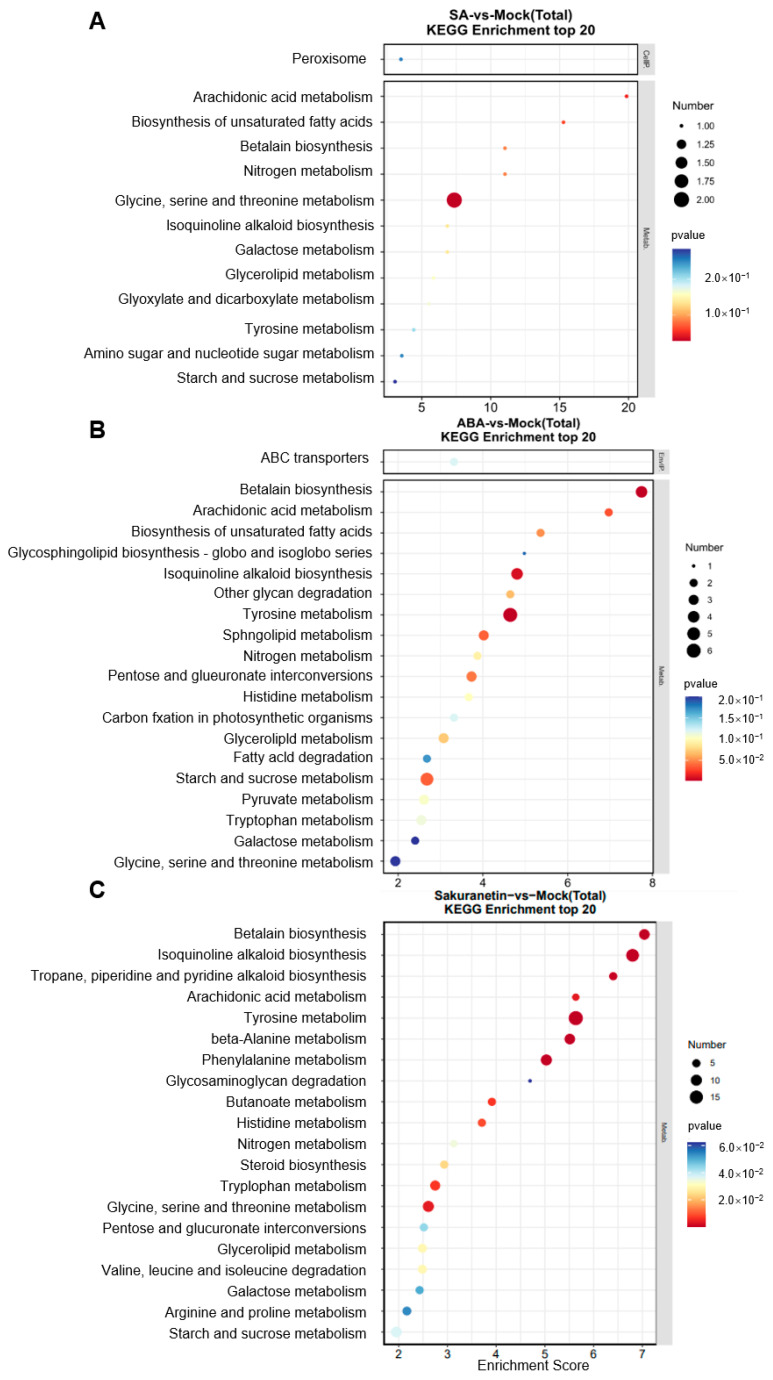
KEGG analysis of the DEGs. (**A**) SA treatment. (**B**) ABA treatment. (**C**) sakuranetin treatment.

To evaluate the signaling pathway of the DEGs, KEGG analysis of the DEGs in three treatment groups was performed. KEGG is typically divided into three levels. Level 1 contains six categories: metabolism, genetic information processing, environmental information processing, cellular processes, organismal systems, and human diseases (species-specific notes may be deleted). Level 2 contains 44 categories, including cell growth and death, transcription, and development. Level 3 is the hundreds of pathways used for routine enrichment. Among all the three groups, metabolism is the pathway containing the most DEGs, and the main enrichment pathway is the carbohydrate, lipid, and amino acid metabolism pathway (Figure 5A–C). In the sakuranetin treatment group, the biosynthesis of other secondary metabolites, metabolism of other amino acids, and energy metabolism pathways are also enriched (Figure 5C). Our data also presented the top 20 items in the KEGG enrichment analysis of these three groups (Figure 6). In terms of amino acid metabolism, the glycine, serine, and threonine metabolism are enriched in the SA treatment group, while the tyrosine metabolism is enriched in the other two groups. Betalain biosynthesis and isoquinoline alkaloid biosynthesis are enriched in both ABA and sakuranetin treatment groups (Figure 6B,C), which suggests these two molecules or the derivates may contribute to the adaptation to the environment.

### 3.6. Quantitative Real-Time PCR Validation

**Figure 7:**
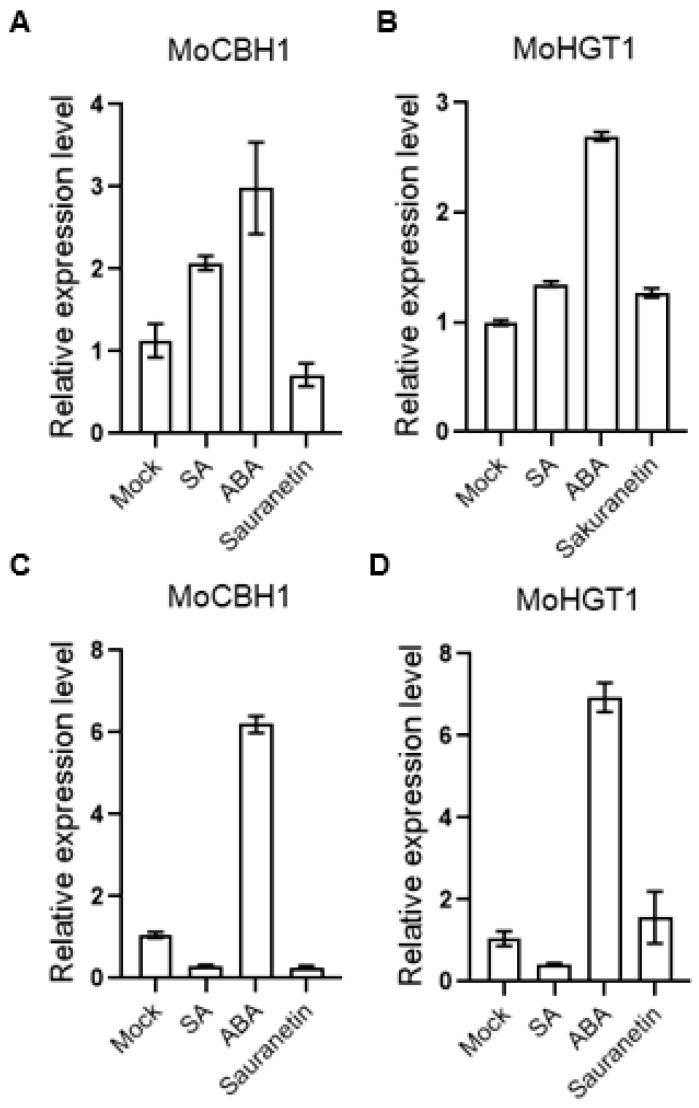
Expression of ABA-induced virulence-related gene. (**A**) The expression of MoCBH1 from transcriptome data. (**B**) The expression of MoHGT1 from transcriptome data. (**C**) The expression of MoCBH1 from *M. oryzae* on the leaves. (**D**) The expression of MoHGT1 from *M. oryzae* on the leaves. Error bars indicate SD of three technical repeats. This is a representative experiment that was repeated three times with similar results.

Since the transcriptome data after treatment of plant metabolites were obtained from *M. oryzae* cultured on the medium, to further verify whether these data were consistent with or close to *M. oryzae*-infected rice, total RNA from the disease spots of rice leaves was extracted, and the expression of some genes was verified by QRT-PCR. Among these differentially expressed genes, we selected genes that changed only after ABA treatment and have GO annotations for validation. According to our transcriptome data, the gene MoCBH1 (MGG_10712), annotated by GO analysis as hydrolase activity, hydrolyzing O-glycosyl compounds and involved in the cellulose catabolic process from GuickGo, was up-regulated by ABA (Figure 7A). MoHGT1 (MGG_08446) is another gene up-regulated by ABA treatment (Figure 7B). GO annotation From QuickGo showed that it has transmembrane transporter activity with transporting carbohydrates. In the results of QRT-PCR from the disease spots of rice leaves, both genes were up-regulated on the ABA-treated *M. oryzae* (Figure 7C,D). These results suggest that our transcriptome data may simulate the gene expression changes during *M. oryzae* infection to some extent.

## 4. Discussion

Fungal diseases are one of the major biotic stresses that hinder rice production and threaten agricultural production. In addition to human intervention in the growing environment of rice to prevent diseases, improving the disease resistance of plants is also an important way to ensure food security. SA is a plant hormone that has been widely studied in relation to plant immunity. The loss of SA will lead to the destruction of the disease resistance of rice. SA 5-hydroxylases can catalyze SA to 2,5-dihydroxybenzoic acid (2,5-DHBA) and decrease the SA level in rice. Studies have shown that the disruption of SA 5-hydroxylases can cause an increase in SA levels in rice and increase broad-spectrum resistance to many diseases, including rice blast [16]. When the rice expressed an SA-degrading hydroxylase, the SA-deficient rice plants are more susceptible to rice blast infection [17]. It is worth noting that SA is defined as a plant hormone rather than a phytoalexin. Its primary function is to transmit plant immune signals rather than as a functional molecule that ultimately acts directly on pathogens. Therefore, this may also explain why SA treatment on the medium did not significantly inhibit the growth of *M. oryzae*. Though SA does not act directly on *M. oryzae*, our transcriptome data show that SA treatment can also cause changes in gene expression in *M. oryzae*. However, fewer genes are affected compared to the other two treatments. Therefore, it cannot be ruled out that SA may weakly or invisibly inhibit the growth of *M. oryzae* directly.

Compared with SA, ABA can induce more gene expression changes after a 10-day treatment. Different from SA and sakuranetin, which can enhance the immunity of rice, ABA has a negative effect on the disease resistance of rice. Moreover, this negative effect on plant immunity affects both plants and pathogens. In plant signaling pathways, ABA can inhibit immune signals transmitted by SA. WRKY45 and OsNPR1 are the two key components in the SA signal. When rice is infected by *M. oryzae*, the expressions of OsNPR1 and WRKY45, which are the receptors of SA and an immunity transcription factor, respectively, are elevated. ABA can markedly suppress the transcriptional up-regulation of these two genes and attenuate the SA-mediated basal immunity in rice [18]. In addition to antagonizing the immune signals of plants, ABA can also directly act on fungi and promote the spore germination and vegetative growth of *M. oryzae* [9]. Moreover, *M. oryzae* can also synthesize ABA to promote its growth and infection in the host, which further indicates the important role of ABA in the virulence of *M. oryzae* [9]. Since the medium used for cultivating *M. oryzae* was already suitable for fungal growth, there was no significant difference in the growth of fungi between the ABA treatment group and the control group. Different from the treatment on the medium, after the treatment of ABA on the leaves, it was observed that the lesion area of *M. oryzae* on the leaves was larger, and the leaves were also significantly yellow compared with other groups. The results further showed that ABA can promote the rice blast infection.

Plants use a variety of strategies to deal with pathogen invasion, including stomatal closure, phytoalexins biosynthesis, cell wall strengthening, slowing down growth, and hypersensitive response cell death [19,20]. Compared with other strategies involving the physiological processes of plants and complex gene regulatory networks, the production of phytoalexins can be simplified to the catalytic reactions of various metabolites in plants. Sakuranetin, as a rice flavonoid phytoalexin, has a relatively simple synthetic pathway, and studies have shown that not only can it help rice effectively resist the infection of rice blast but also resist the invasion of brown planthopper [21,22]. Though sakuranetin is effective against the invasion of pathogens, the molecular mechanism of inhibiting the growth and infection of *M. oryzae* is still unclear. Our results showed that hyphae expansion of the fungus was inhibited on media containing sakuranetin. The effects of sakuranetin on hyphae and spore morphology can be further observed by electron microscopy in future studies.

A study has shown that some species of rice blast fungus can metabolize sakuranetin into sternbin and naringenin, thereby alleviating phytoalexin toxicity [23]. Therefore, searching for the molecular mechanism of sakuranetin’s inhibition of *M. oryzae* not only helps to understand the target of phytoalexin but also helps to develop new artificial phytoalexins in the future to prevent and control pathogens while avoiding the evolution of drug resistance. However, in the infection experiment on the rice leaves, sakuranetin treatment did not show a significant inhibition of the fungal invasion (Figure 2), which was different from the results of previous studies [13]. We hypothesize that this is due to different culture conditions. Compared with rice leaves, the environment on the medium lacks the interference of plant complex physiological reactions and metabolites. Once the fungi invade the rice cell, the cell wall and membrane may act as a barrier to protect the fungi from the extracellular phytoalexin. Although the mechanism of inhibiting *M. oryzae* by sakuranetin is not clear, we hypothesize that it may act directly on hyphae and inhibit its growth. Under natural conditions, sakuranetin is not released outside the cell but plays a role in the cell, which explains why in vitro treatment of sakuranetin did not significantly inhibit the *M. oryzae* infection. In our transcriptome data, thousands of the *M. oryzae* genes’ expressions were changed by sakuranetin treatment. However, the genes of *M. oryzae* are not as well studied as those of other model organisms, and many differentially expressed genes in our transcriptome data are not annotated. It is possible that these unannotated genes were the direct targets of sakuranetin. The future development of new techniques to study the interaction of molecules with proteins of unknown function will help to reveal the mechanism of various phytoalexins.

In this study, we identified two ABA-specific up-regulated genes, MoCBH1 (MGG_10712) and MoHGT1 (MGG_08446), from the transcriptome data, and both genes were also up-regulated by ABA during *M. oryzae* infection verified by QRT-PCR. Cellobiosidase is one of the cell wall-degrading enzymes. Research on another rice pathogen, *Xanthomonas oryzae pv. oryzae,* which causes bacterial blight of rice, showed that the cellobiosidase is critical for virulence [24]. As an important enzyme for cellulose degradation, no cellobiosidase from *M. oryzae* has been studied in detail. Carbohydrate transporters play an important role in nutrient absorption by pathogens. The biotrophic fungus *Ustilago maydis* causes corn smut disease. A study found that the fungal sucrose transporter UmSrt1 can help *U. maydis* compete for extracellular sucrose, which can promote its growth and infection [25]. However, until now, only two sugar transporters localized on the endoplasmic reticulum of *M. oryzae* have been reported [26], and the carbohydrate transporters localized on the cell membrane have not been studied. According to our data, these two *M. oryzae* genes are up-regulated by ABA both on the medium and in rice leaves and combined with the studies of other pathogens. These two genes may play a positive role in the virulence of rice blast.

There are thousands of plant metabolites in rice. SA, ABA, and sakuranetin are only a few metabolites that have been studied. Though the effects of other metabolites may not be as obvious as these widely studied substances, they may also have potential roles in plant growth or disease control. *M. oryzae* is a kind of plant pathogenic fungi that has been studied widely. The analysis of transcriptome after treatment of plant metabolites will also provide a reference for the control of other plant pathogenic fungi that have been studied less.

## 5. Conclusions

In this study, we treated the rice pathogenic fungi *M. oryzae* with SA, ABA, or sakuranetin. On the medium, the plant hormones SA and ABA did not show a significant effect on the growth of *M. oryzae*. In contrast, the plant phytoalexin sakuranetin severely inhibited the growth of *M. oryzae*. In the experiment of rice blast inoculation, ABA treatment can significantly increase the infection of pathogens. The total RNA of *M. oryzae* on different treatment mediums was extracted, and the transcriptomes were analyzed. Additionally, 185 and 38 genes, 803 and 156 genes, and 1525 and 428 genes were up- or down-regulated after SA, ABA, or sakuranetin treatment. According to GO analysis, most DEGs were annotated to the cellular process and metabolic process in the biological process category and binding and catalytic activity in the molecular function category. By KEGG pathway analysis, metabolism is the pathway containing the most DEGs, and the main enrichment pathway is the carbohydrate, lipid, and amino acid metabolism pathway. From the transcriptome data, we found two genes, MoCBH1 (MGG_10712) and MoHGT1 (MGG_08446), were up-regulated by ABA treatment, and similar expressions were also presented in the *M. oryzae*-infected rice leaves verified by QRT-PCR.

## Data Availability

Data are contained within the article.

## Supplementary Materials

The following supporting information can be downloaded at https://www.mdpi.com/article/10.3390/pathogens13020105/s1. Table S1: Primers used for quantitative real-time PCR. Table S2: The gene expression data in this study. Table S3: The annotation information of genes from QuickGo. Table S4: The genes’ expression changes of ABA treatment vs. mock treatment. Table S5: The genes’ expression changes of SA treatment vs. mock treatment. Table S6: The genes’ expression changes of sakuranetin treatment vs. mock treatment.
